# Inulin and multispecies probiotic effects on blood, liver and kidney biochemistry and metabolic and stress-related gene expression in pigs

**DOI:** 10.1038/s41598-026-43434-7

**Published:** 2026-03-13

**Authors:** Adam Lepczyński, Agnieszka Herosimczyk, Małgorzata Ożgo, Aleksandra Dunisławska, Michalina Adaszyńska-Skwirzyńska, Anna Tuśnio, Elżbieta Pietrzak, Adrianna Konopka, Kamil Gawin, Ewa Święch, Elżbieta Redlarska, Sylwia Słuczanowska-Głąbowska, Marcin Taciak, Mateusz Bucław, Łukasz Struk, Marcin Barszcz

**Affiliations:** 1 Department of Physiology, Cytobiology and Proteomics, Faculty of Biotechnology and Animal Sciences, West Pomeraninan University of Technology in Szczecin, Janickiego 29, 71-270 Szczecin, Poland; 2 Department of Animal Biotechnology and Genetics, Faculty of Animal Breeding and Biology, Bydgoszcz University of Science and Technology, Mazowiecka 28, 85-084 Bydgoszcz, Poland; 3 Department of Monogastric Animal Sciences, Faculty of Biotechnology and Animal Sciences, West Pomeranian University of Technology in Szczecin, Janickiego 29, 71-270 Szczecin, Poland; 4 Department of Animal Nutrition, The Kielanowski Institute of Animal Physiology and Nutrition, Polish Academy of Sciences, Instytucka 3, 05-110 Jabłonna, Poland; 5 Laboratory of Analysis of Gastrointestinal Tract Protective Barrier, Department of Animal Nutrition, The Kielanowski Institute of Animal Physiology and Nutrition, Polish Academy of Sciences, Instytucka 3, 05-110 Jabłonna, Poland; 6 Department of Physiology, Pomeranian Medical University in Szczecin, Powstańców Wielkopolskich 72, 70-111 Szczecin, Poland; 7 Department of Animal Breeding and Nutrition, Faculty of Animal Science, Institute of Animal Sciences, Warsaw University of Life Sciences, Ciszewskiego 8, 02-786 Warsaw, Poland; 8 Department of Organic Chemistry and Physical Chemistry, Faculty of Chemical Technology and Engineering, West Pomeranian University of Technology in Szczecin, Piastów 42, 71-065 Szczecin, Poland

**Keywords:** Monogastric, Prebiotic, Probiotic, Metabolism, One Health, Biochemistry, Immunology, Physiology

## Abstract

Dietary supplementation with probiotics, prebiotics, and their combination (synbiotics) can improve pig health through direct and indirect mechanisms. This study evaluated the effects of probiotics, inulin, and their combination on: (1) blood biochemistry parameters reflecting lipid metabolism, liver function, oxidative stress, and immune status; (2) liver and kidney gene expression and biochemical markers related to energy and reactive oxygen species metabolism; and (3) mineral profiles in blood plasma, liver, and kidneys, including renal aquaporin gene expression. Inulin increased total protein, while raising total and LDL-cholesterol but lowering hepatic cholesterol and triglyceride levels, and upregulating apolipoprotein A1 expression. Probiotics lowered AST and ALT activity and enhanced the expression of energy metabolism genes in the liver and kidneys, similarly to the combined treatment. Inulin increased plasma sodium and phosphorus but reduced liver magnesium and copper contents; probiotics elevated selenium, iron, and phosphorus concentrations in the blood and kidneys. Both supplements improved antioxidant capacity and anti-inflammatory responses, although their combination unexpectedly elevated renal interleukin-6 expression. Overall, probiotics, inulin, and their combination positively affected lipid metabolism, liver function, oxidative balance, and immune status, supporting their potential as natural alternatives to antibiotics in pig nutrition.

**Supplementary Information:**

The online version contains supplementary material available at 10.1038/s41598-026-43434-7.

## Introduction

Inulin-type fructans (ITFs) are linear oligomers and polymers of fructose units linked by β(2 → 1) glycosidic bonds that resist digestion by small intestinal enzymes. Typically, the chain is terminated by a glucose unit linked through an α(1 → 2) bond. Inulin is a plant storage polysaccharide present in many species; however, for industrial purposes, it is mainly extracted from chicory (*Cichorium intybus* L.) root^1,2^. Native chicory inulin contains approx. 10% oligofructose and has an average degree of polymerisation (DP) of 10–12. Removing this short-chain fraction allows to obtain longer-chain inulin with an average DP of 25^1,3^. The DP determines inulin’s biological activity in the gut^4–8^, although, its effects extends beyond the intestine. Inulin supplementation has been shown to improve bone health in growing pigs fed a high-fat diet rich in saturated fatty acids^9^ and exert antioxidant effects while modulating gene and protein expression in the liver, including those involved in lipid metabolism^10,11^. Inulin also affects kidney physiology in pigs by increasing the expression of Mg^2+^-specific cation channels involved in magnesium homeostasis^12^ and modulates the expression of heat shock, cytoskeletal, and immune-related proteins, as well as those involved in cellular respiration, glycolysis and gluconeogenesis^13^. Inulin-type fructans also modify the mineral composition of blood plasma^14^, liver and kidneys^15,16^.

As a feed additive, inulin is often combined with probiotic bacterial and yeast strains to increase their health-promoting effects^17–20^. The efficacy of such a preparation can be further improved by incorporating a multispecies microbial consortium, where individual organisms show complementary fermentation capabilities or target different pathogens^21–23^. In our previous research on piglets, we applied a probiotic composed of *Lactococcus lactis*, *Carnobacterium divergens*, *Lactobacillus casei*, *Lactobacillus plantarum* and *Saccharomyces cerevisiae*^17^*. Lactococcus lactis* is a Gram-positive lactic acid bacteria belonging to the *Streptococcus* family. It is a facultative anaerobe producing L-lactic acid through glycolysis, and synthesises nisin A, a bacteriocin with antimicrobial activity against *Bacillus cereus*, *Listeria monocytogenes*, *Clostridium botulinum* or enterococci^24^. *Carnobacterium divergens* has similar characteristics but shows greater metabolic adaptability and environmental tolerance. It is generally homofermentative but can also generate products typical of heterofermentative metabolic pathways^25^. *Lactobacillus casei* is a facultatively heterofermentative species that stimulates goblet and Paneth cell activity, as well as the expression of tight junction proteins and mucin gene expression, helping maintain intestinal barrier integrity^26^. *Lactobacillus plantarum* is an anaerobe or facultative anaerobe widely used as a probiotic in food and medicine. It is common in fermented foods and the gastrointestinal tract and exerts antibacterial, antifungal, and potential therapeutic effects in hypertension, diabetes, obesity, and certain cancers^27^. The yeast *Saccharomyces cerevisiae* improves feed efficiency, animal health, and reduces pathogen load and the environmental impact of livestock production. It is rich in protein, vitamins and minerals, and contains mannoproteins and β-D-mannan with immunomodulatory properties^28^.

Multispecies probiotic formulation may support antioxidant defence mechanisms^29^, and lower plasma cholesterol (TC) and triglyceride (TG) levels^30^. Recent studies on human have indicated a close relationship between metabolic disorders and the intestinal microbiota. Dietary supplementation with probiotics improved hepatocyte condition and reduced liver enzyme activity, as well as concentrations of triglycerides and low- and high-density lipoproteins in patients with non-alcoholic fatty liver disease^31^. A multispecies probiotic composed of *L. plantarum*, *L. fermentum* and *L. reuteri* positively affected liver function and reduced uric acid level in the blood of patients with this metabolic disorder^32^. Probiotics have also been used in the treatment of chronic kidney disease^33^, and a meta-analysis revealed that these supplements could improve kidney function, glucose and lipid metabolism, and reduce inflammation and oxidative stress in human patients with diabetic kidney disease^34^.

The findings collectively suggest that inulin and probiotics can significantly influence liver and kidney physiology. We hypothesised that these dietary supplements would reduce oxidative stress, regulate lipid metabolism, and modify the mineral composition of blood, liver and kidneys of pigs. Therefore, the aim of the study was to determine the effect of inulin and a multispecies probiotic on biochemical indices and mineral profiles in these tissues, as well as on the expression of genes related to energy metabolism and oxidative stress protection in pigs.

## Methods

### Study design

The experimental procedures, animal housing and performed analyses are in accordance to ARRIVE guidelines.

The experimental protocol was approved by the Third Local Animal Experimentation Ethics Committee (permission No. 30/2010, Warsaw University of Life Sciences-SGGW, Warsaw, Poland) according to the principles of the European Union and Polish Animal Protection Act.

The experiment involved 32 castrated male piglets (DanBred x Duroc) obtained from a commercial farm located in Bełżyce, Poland. Animals were divided into four groups (n = 8) fed the following diets: control diet, control diet with 0.05% addition of probiotics, diet with 2% of inulin, and diet with 2% of inulin and 0.05% of probiotics. The feed additives used in the trial were: Inulin Orafti®HPX (BENEO GmbH, Mannheim, Germany) with an average degree of polymerization of 23 and multispecies probiotic of JHJ Ltd. (Gizałki, Poland) containing min 10^7^ cfu/g of *Saccharomyces cerevisiae* ŁOCK 0141 and min 10^9^ cfu/g of each bacteria: *Lactococcus lactis* IBB500, *Carnobacterium divergens* S1, *Lactobacillus casei* ŁOCK 0915, and *Lactobacillus plantarum* ŁOCK 0862. Diets were cereal-based, isoprotein and isoenergetic, and their composition was published by Barszcz et al.^17^. The mineral composition of the diets is provided in the Supplementary Table 1.

The housing conditions were also described previously by Barszcz et al.^17^. Briefly, at the beginning of the experiment the piglets were kept with their sows (four litters per group) in a farrowing pens on the farm. From the 10^th^ day of life piglets were offered the experimental diets but sows’ milk was their main feed until weaning at 28^th^ day of age. On that day, the animals were weighed, ear-tagged and two barrows from each litter were chosen and transported to the experimental unit. The average body weight of the selected piglets was 7.9 kg. Then, the piglets were divided according to dietary treatment and placed in pens of four animals each (two pens per group). Animals had unlimited access to feed and water during the whole experimental period. The housing conditions were as follows: temperature 25 °C, 12 dark/12 light cycle, and humidity 55%. After 40 days of the feeding trial, at 50 day of age, pigs were stunned by electric shock and exsanguinated. For biochemical analyses mixed blood was taken from jugular veins and carotid arteries into heparinised tubes and centrifuged (10 min, 1500 g, 4 °C). Plasma was stored at (− 40)°C until further analyses. The abdominal cavity was opened and liver and kidneys were excised. The samples of internal organs were taken, snap-frozen in liquid nitrogen and stored at (− 80)°C. The experiment was conducted from December 2013 to February 2014.

### Biochemical analyses of blood plasma and organs

Basic biochemical parameters of blood plasma were analyzed spectrophotometrically using the following ready-to-use reagents of ELITech Clinical Systems SAS (Sees, France): ALBUMIN (catalogue No. ALBU-0700), ALP (DEA) SL (PASL-0230), ALT/GPT 4 + 1 SL (ALSL-0430), AMYLASE SL (AMSL-0230), AST/GPT 4 + 1 SL (ASSL-0430), BILIRUBIN TOT 4 + 1 (BITO-0600), CALCIUM ARSENAZO (CALA-250), CHLORIDE (CHLO-0600), CHOLESTEROL SL (CHSL-0507), CHOLESTEROL HDL SL 2G (HDLL-0230), CHOLESTEROL LDL SL 2G (LDLL-0230), CHOLINESTERASE (CHES-0053), CK NAC SL (CKSL-0230), CREATININE JAFFE (CRCO-0600), GAMMA GT SL (GISL-0420), GLUCOSE PAP SL (GPSL-0700), IRON CHROMAZUROL (FECA-0600), LDH-L SL (LLSL-0400), LIPASE SL (LPSL-0230), MAGNESIUM CALMAGITE (MAGN-0600), PHOSPHORUS (PHOS-0600), TOTAL PROTEIN PLUS (PROB-0600), TRIGLYCERIDES MONO SL NEW (TGML-0517), UREA UV SL (URSL-0400), URIC ACID MONO SL (AUML-0420). Fibrinogen level was measured based on a clotting time using the diagnostic kit Dia-Fib (cat. No. 61024) of DIAGON Ltd. (Budapest, Hungary), while immunoglobulin A, G and M concentrations were determined by the immunoturbidimetry using specific antisera and buffers of APTEC Diagnostics nv (Sint-Niklaas, Belgium) of the following cat. No.: IIGA-0400, IIGG-0400, and IIGM-0400. All measurements were performed on a Maxmat PL multidisciplinary diagnostic platform (Erba Diagnostics SARL, Montpellier, France) based on the manufacturers protocols.

For triglyceride and cholesterol analyses, frozen liver and kidney samples (2.0 g) were homogenized in 4 ml of ice-cold 50 mM/l 1,4-piperazinediethanesulphonic acid buffer (pH 7.0), and centrifuged for 30 min at 12,850 g at 4 °C. The supernatants were used for the direct analyses. Both cholesterol and triglyceride concentrations were measured using the diagnostic kits of ELITech Clinical Systems SAS based on the manufacturer protocol using Maxmat PL analyzer.

For analyses of thiobarbituric acid-reactive substances (TBARS), prooxidants-antioxidants balance (PAB) and antioxidant enzymes 2 g liver and kidney pieces were homogenized in 2 ml and 4 ml, respectively, of ice-cold 0.9% NaCl and centrifuged at 10,000 g for 10 min at 4 °C. The concentration of TBARS in the supernatants, as well as in blood plasma, was measured colourimetrically as described by Chodkowska et al.^35^ For the purpose of this analysis the following reagents were used: trichloroacetic acid, cat. No. 577970115 (POCH S.A., Gliwice, Poland), 2-thiobarbituric acid, cat. No. T5500 (Sigma-Aldrich, Inc., St. Louis, MO, USA) and malondialdehyde standard, cat. No. 10009202 (Cayman Chemical, Ann Arbor, MI, USA). PAB was measured according to the microplate method of Koliakos and Hamidi Alamdari^36^ using the following reagents: 3,3′,5,5′-tetramethylbenzidine, cat. No. T5525, ammonium persulphate, cat. No. A3678, horseradish peroxidase, cat. No. P8375, uric acid, cat. No. U0881 (Sigma-Aldrich, Inc.), and hydrogen peroxide, cat. No. 885193111 (POCH S.A.). Activities of catalase (CAT), glutathione peroxidase (GSHPX) and superoxide dismutase (SOD) (total, MnSOD, and CuZnSOD) were determined spectrophotometrically using the microplate methods described previously by Wypych et al.^37^ For these analyses the following diagnostic kits and reagents were used: Catalase Assay Kit, cat. No. K-CATAL (Megazyme, Bray, Ireland), Glutathione Peroxidase (GSH-PX) Assay Kit, cat. No. SH0028 (BT LAB, Shanghai, China), Trizma Base, cat. No. T1503, diethylenetriaminepentaacetic acid, cat. No. D6518, potassium cyanide, cat. No. 60178, pyrogallol, cat. No. P0381 (Sigma-Aldrich, Inc.). Total protein concentration in liver and kidney samples was measured using the Bio-Rad Protein Assay Kit II, cat. No. 5000002 (Bio-Rad, Hercules, CA, USA). All measurements of absorbance were performed on a SpectraMax iD3 multi-mode microplate reader (Molecular Devices, LLC., San Jose, CA, USA).

### Blood plasma, kidney and liver mineral content

The mineral composition of the samples was determined according to the methodology published by Adaszyńska-Skwirzyńska et al.^38^. Selected minerals in liver and kidney were quantified by inductively coupled plasma optical emission spectrometry (ICP-OES) using an Optima 2000DV instrument (Perkin Elmer, Waltham, USA). Frozen samples (700 mg) were microwave-digested using a Speedwave Xpert oven (Berghof GmbH, Eningen, Germany) with 8.0 mL of 69% HNO_3_ and 2.0 mL of 30% H_2_O_2_ (Supelco, Bellefonte, USA) for 15 min before sealing. Calibration standards (0.1–150 mg/L) were prepared from a TraceCert® multi-element standard solution (Sigma-Aldrich, St. Louis, USA) with yttrium (Merck, Darmstadt, Germany) applied as internal standard. Mineralized solutions were diluted directly before analysis. The measurement results were analyzed based on standard curves plotted for each of the elements. All analyses were performed in triplicate, with quality control utilizing certified bovine muscle reference material (NIST 8414; Gaithersburg, Maryland, USA). The results of the reference material analyses are shown in the Supplementary Table 2.

### Quantitative real-time PCR

RNA isolation from porcine liver and kidney samples previously fixed in stabilizing buffer (Fix RNA, EURx, Gdańsk, Poland) was performed using RNA Extracol reagent (EURx, Gdańsk, Poland) and a commercially avaliable RNA purification kit (Universal RNA Purification Kit, EURx, Gdańsk, Poland). Both organ samples (n = 4/group) were processed using a TissueRuptor homogenizer (Qiagen GmbH, Hilden, Germany) in RNA Extracol reagent and purified on silica columns. The RNA quality was examined using agarose gel electrophoresis (2%) and its quantity was measured spectrophotometrically (UV–Vis NanoDrop 2000c/2000, Thermo Fisher Scientific Nanodrop Products, Wilmington, NC, USA). Reverse transcription of mRNA into cDNA was performed using the smART First Strand cDNA Synthesis kit (Universal RNA Purification Kit, EURx, Gdańsk, Poland), according to the manufacturer’s instructions. The synthesized cDNA was used as a template for quantitative polymerase chain reaction (qPCR). The qPCR reaction mixture contained SG qPCR Master Mix (2x) (EURx, Gdańsk, Poland), 1 μM of each oligonucleotide primer, and diluted cDNA (140 ng). The thermal cycling was conducted using a LightCycler 480 II (Roche Diagnostics, Basel, Switzerland) according to Beldowska et al.^39^. Each RT-qPCR reaction was performed in two technical replicates. In the liver, the relative gene expression analysis was performed for four panels of genes: lipid and steroid metabolism *(HSD3B, APOA1, BAAT, CYP1A2, HMGCR, MVK, PPARA, PPARG*), transport and metabolic regulation (*ABCC4, PRKAA1, TFRC*), oxidative stress and antioxidants (*CAT, GPX1, GPX4, PRDX6, SELENOP, SOD1, TXNRD1*), and immune response (*CRP, IL6*). In the kidney, the analysis was performed for: lipid and steroid metabolism (*HSD3B, APOA1, CYP1A2, HMGCR, MVK, PPARA, PPARG*), transport and metabolic regulation (*ABCC4, AQP2, AQP3, PRKAA1, TFRC*), oxidative stress and antioxidants (*CAT, GPX1, GPX4, PRDX6, SELENOP, SOD1, TXNRD1*), and immune response (*CRP, IL6*). The primer sequences are provided in Table 1. Primer annealing to the cDNA template for each gene was performed at 58 °C. At the end of the reaction, a melting curve was generated to verify the specificity of the amplicon. Primer sequences were designed using Primer-BLAST available at NCBI (https://www.ncbi.nlm.nih.gov/tools/primer-blast/). Relative gene expression was calculated separately for each experimental group using the ΔΔCt method^40^. The geometric mean of the threshold cycle (Ct) values for beta-actin (*ACTB*) and ribosomal protein L4 (*RPL4*) was used as an internal control for the calculations.

**Table 1:** Primer sequences used in RT-qPCR analysis (F—forward primer; R—reverse primer).

Gene	Name	NCBI no.	Primer sequences
Reference genes			
*RPL4*^*1*^	ribosomal protein L4	100038029	F: CAAGAGTAACTACAACCTTC R: GAACTCTACGATGAATCTTC
*ACTB*^*1*^	beta-actin	41396	F: CACGCCATCCTGCGTCTGGA R: AGCACCGTGTTGGCGTAGAG
Lipid and steroid metabolism			
*HSD3B*	3β hydroxysteroid dehydrogenase	445539	F: CAAAAATCCCATGTGCCCCC R: AAGCTCCATCCATTCCTGCC
*APOA1*	Apolipoprotein A1	397691	F: AATTCTGCTCCCCATGCTCAG R: TCTTCTCCTCCAGGTCACCCA
*BAAT*	Bile acid-CoA:amino acid N-acyltransferase	102158534	F: TGGATAGGGCCAGTCTTTCT R: GCAGCAGCTTGCTTCCAAAA
*CYP1A2*	Cytochrome P450 family 1 subfamily A member 2	100152910	F: GCACTTGCACATGTTTTCTGC R: CAGCCCCTATTCTGTGAGGC
*HMGCR*	HMG-CoA reductase—3-hydroxy-3-methylglutaryl-coenzyme A reductase	100144446	F: TGTGTGCGGTACAGTGATGG R: AGTCTTGAAGGCTTACCTGTTGT
*MVK*	Mevalonate kinase	100152230	F: AAGAGGCAGCGACCATTCAG R: TCACTGGGGATGGAAGAGCTA
*PPARA*	Peroxisome proliferator activated receptor alpha	397239	F: GCATTTAGAGGCGTGGCATC R: TCTCAGTTGGGGAGCCTCTT
*PPARG*	Peroxisome proliferator activated receptor gamma	397671	F: GCTTCTCTCACCCGAAAGTG R: CGTGTCCCAAGATGTGTTTG
Oxidative stress and antioxidants			
*CAT*	Catalase	397568	F: CTATCGGATTCCCAGAGCCG R: AACGAGGTACCCTCTGACCT
*GPX1*	Glutathione peroxidase 1	397403	F: TAGCTTGCACAACCTCAGGG R: CGGGAGTGAGACTGGGGTTA
*GPX4*	Glutathione peroxidase 4	399537	F: CCTTGGCTGAGAATTCGTGC R: CTGGGTTGAGAGAGTTGCGG
*PRDX6*	Peroxiredoxin 6	399538	F: AAAAGAGGGTTGCCACTCCG R: CTGCATGCCAGAAACACAGC
*SELENOP*	Selenoprotein P	100037964	F: GCATGGGGTCTTGGTCCTTT R: TCTTTTCCAGGACGACCCCA
*SOD1*	Superoxide dismutase 1	397036	F: CATTCCATCATTGGCCGCAC R: TGGGGACCTTTAGAAACCAGG
*TXNRD1*	Thioredoxin reductase 1	396681	F: CCTCGACGTTCCACCCATAG R: CTTCTCAATGCCGTTGCAGG
Transport and metabolic regulation			
*ABCC4*	ATP-binding cassette sub-family C member 7	100152536	F: TTTTGTTCTGCGCATCTGTC R: TTGCCAGCCTTTGAGTTCTT
*AQP2*	Aquaporin 2	100147810	F: GCACAGACCTTTCCCCAAAGA R: ATCTGCCCCTTGGTGGTATC
*AQP3*	Aquaporin 3	100126235	F: GGCCCTAACCACATCTGTCTG R: TGGGACAGCTTCACATTCTCC
*PRKAA1*	Protein kinase AMP-activated catalytic subunit alpha 1	100145903	F: CCTGTGGGCTCTACAGCAAT R: ACTAACTGAGCCTTGCTCTATCA
*TFRC*	Transferrin receptor	397062	F: TGGCTGTATTCTGCTCGTGG R: TGAGTTCCTTCTCTTCTGAGGG
Immune response			
*CRP*	C-reactive protein	396842	F: AGCTACTGCTGCTCTTTCCA R: GCACAGGCAACCTTGATACC
*IL6*	Interleukin-6	399500	F: CCACCGGTCTTGTGGAGTTT R: TTGCACAGTCGGGTTGTCTA

^41^(Nygard et al., 2007).

### Statistical analysis of data

Statistical analysis was performed based on normalized ΔCt data (Ct of target gene $-$ geometric mean Ct of *ACTB* and *RPL4*). Data were tested for normality (Shapiro–Wilk test) and homogeneity of variance (Levene’s test). Based on the results, a non-parametric Kruskal–Wallis test was applied. When the experimental factor showed a statistically significant effect (*p* ≤ 0.05), a post-hoc Dunn’s multiple comparison test was conducted. Due to the low number of biological replicates, for genes where the Kruskal–Wallis test showed 0.05 < *p* < 0.099, an uncorrected Dunn’s test with lower sensitivity was performed to indicate trends. All calculations and graphical presentation of gene expression were prepared in the GraphPad Prism 9 for Windows (GraphPad Software, San Diego, California USA). The results of biochemical and chemical analyses of blood, liver and kidney were submitted to two-way analysis of variance followed by Fisher’s least significant difference test using the Statgraphics Centurion XVI ver. 16.1.03 statistical software (StatPoint Technologies, Inc., Warrenton, VA, USA). The statistical analyses were performed according to Dawson and Trapp^42^.

## Results

### Liver function markers

Feeding an inulin-enriched diet significantly increased plasma total protein levels, while probiotic supplementation markedly altered plasma AST activity. A significant interaction between probiotic and prebiotic was also recorded for plasma AST and ALT activities, as well as urea concentration (Table 2). In pigs fed the control diet, probiotic supplementation reduced AST and ALT activities compared with the unsupplemented control group, whereas in animals receiving inulin-enriched diet, the probiotic had no effect on these enzymes.

**Table 2:** Blood plasma biochemical parameters in pigs fed the control diet (C) or diets supplemented with inulin (Inu), probiotic (Pro) or inulin with probiotic (Com).

	Group					*p*-value		
	C	Pro	Inu	Com	SEM	Probiotic	Inulin	Interaction
Total protein, g/L	43	42	45	44	0.5	0.2657	**0.0414**	0.8947
Albumin, g/L	31	32	32	32	0.6	0.9188	0.7988	0.6469
Fibrinogen, g/L	0.5	0.4	0.9	0.7	0.08	0.3555	**0.0244**	0.7365
Cholesterol, mmol/L	2.09	2.05	2.22	2.37	0.051	0.5833	**0.0299**	0.3214
HDL-C mmol/L	0.81	0.80	0.76	0.86	0.025	0.416	0.8719	0.3343
LDL-C mmol/L	0.94	0.99	1.15	1.15	0.037	0.7335	**0.0156**	0.7223
TG, mmol/L	0.59	0.6	0.61	0.59	0.023	0.9221	0.8809	0.6819
Glucose, mmol/L	8.63	8.32	8.68	8.39	0.146	0.3251	0.8433	0.9704
AST, U/L	60	41	50	50	2.2	**0.0267**	0.9311	**0.0240**
ALT U/L	75	52	63	69	2.9	0.0963	0.5846	**0.0072**
ALP, U/L	779	695	686	758	32.4	0.9259	0.8162	0.2505
GGT, U/L	28	26	28	29	0.1	0.9055	0.6129	0.5121
Total bilirubin, µmol/L	9.92	9.74	8.69	10.31	0.687	0.6177	0.8190	0.5325
Amylase, U/L	1668	1252	1384	1452	90.6	0.3484	0.8181	0.1939
Cholinesterase, U/L	643	677	716	679	26.8	0.9768	0.5095	0.5230
CK, U/L	1615	663	971	749	165.3	0.0753	0.3876	0.2610
LDH, U/L	594	514	555	517	19.6	0.1448	0.6534	0.5937
Lipase, U/L	4.54	4.25	4.76	3.84	0.441	0.5234	0.9227	0.7367
Creatinine, µmol/L	121.04	115.6	122.08	113.99	2.808	0.2506	0.9610	0.8199
Uric acid, µmol/L	24	11	18	21	2.5	0.2987	0.6951	0.1441
Urea, mmol/L	0.77	1.8	1.26	0.78	0.13	0.224	0.2452	**0.0019**
TBARS, μmol/L	0.81	0.67	0.96	0.62	0.039	**0.001**	0.4974	0.125
PAB, HKU/Ml	150	137	140	130	3.1	0.0523	0.1705	0.8127
Catalase, U/Ml	19.19	11.19	8.94	8.77	1.099	**0.0247**	**0.0010**	**0.0308**
Total SOD, U/mlL	48.88	42.25	43.25	48.38	2.384	0.8792	0.9596	0.2397
GSPX, U/Ml	250	271	258	266	5.7	0.2334	0.8989	0.5757
IgA g/L	1.18	1.08	0.98	0.87	0.057	0.3622	0.0693	0.9605
IgM g/L	1.29	1.20	1.24	1.25	0.024	0.4048	0.8791	0.3298
IgG g/L	8.01	8.25	8.45	8.25	0.127	0.9379	0.4054	0.4148

*HDL-C* high density lipoprotein cholesterol, *LDL-C* low density lipoprotein cholesterol, *TG* triglycerides, *AST* aspartate aminotransferase, *ALT* alanine aminotransferase, *ALP* alkaline phosphatase, *GGT* gamma-glutamyl transferase, *CK* creatine kinase, *TBARS* thiobarbituric acid reactive substances, *PAB* prooxidants-antioxidants balance, *HKU* Hamidi-Koliakos units,  *SOD* superoxide dismutase, *GSHPX* glutathione peroxidase, *C* control group, *Pro* probiotic supplemented group; *Inu* inulin supplemented group, *Com* inulin and probiotic supplemented group.

Significant values are in bold.

### Energetic metabolism

**Fig. 1:**
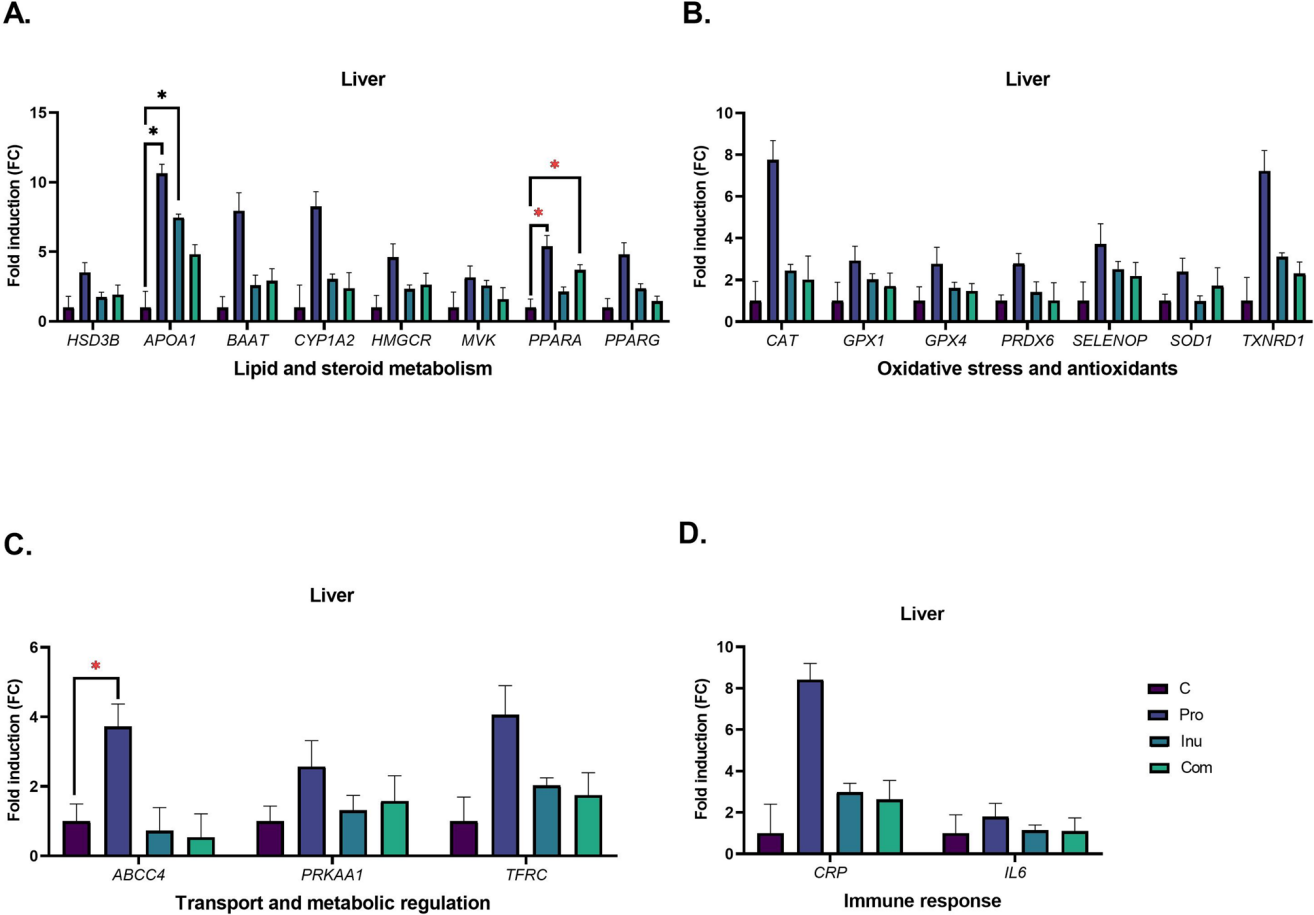
Expression of genes related to energy metabolism, stress response, and cellular defence in the liver of growing pigs fed diets supplemented with probiotic (Pro), prebiotic (Inu) or their combination (Com). Data are presented as mean ± SEM. A black asterisk (*) indicates a significant difference between groups (*p* ≤ 0.05). A red asterisk denotes a statistical trend (0.05 < *p* < 0.099).

Inulin supplementation increased plasma total and LDL-cholesterol (LDL-C) concentrations (Table 2) and reduced total cholesterol (TC) and triglyceride (TG) levels in the liver and kidneys of growing pigs, respectively (Table 3). Moreover, it also upregulated hepatic apolipoprotein A1 (*APOA1*) gene expression compared to the control group (Fig. 1).

**Table 3:** Biochemical parameters of the liver and kidneys in pigs fed the control diet (C) or diets supplemented with inulin (Inu), probiotic (Pro) or their combination (Com).

	Group					*p*-value		
	C	Pro	Inu	Com	SEM	Probiotic	Inulin	Interaction
Liver								
Cholesterol, µmol/g	1.75	1.09	1.31	2.62	0.147	0.1607	**0.0226**	**0.0002**
TG, µmol/g	2.88	2.06	1.95	2.44	0.110	0.3771	0.1482	**0.0014**
PAB, HKU/g	10,221	9409	10587	9778	262.1	0.1367	0.4939	0.9993
TBARS, nmol/g	35.72	39.44	15.88	35.13	4.763	0.0751	0.0658	0.7579
Catalase, U/mg protein	999	874	458	455	78.8	0.6455	**0.0017**	0.6623
Total SOD, U/mg protein	76.17	74.99	71.89	77.6	2.254	0.6362	0.8618	0.4729
Mn SOD, U/mg protein	43.63	50.24	47.16	45.78	1.425	0.3714	0.8727	0.1766
Cu–Zn SOD, U/mg protein	32.54	24.75	24.74	31.82	1.752	0.9189	0.9157	**0.0391**
GSHPX, U/mg protein	21.56	23.71	20.5	18.76	1.039	0.9235	0.1646	0.3624
Kidney								
Cholesterol, µmol/g	2.13	2.07	2.08	2.49	0.122	0.4931	0.4634	0.3586
TG, µmol/g	8.63	5.74	4.75	5.33	0.542	0.2486	**0.0378**	0.0891
PAB, HKU/g	35,812	37,709	35,950	37,346	465.8	0.0947	0.9063	0.7935
TBARS, nmol/g	9.21	5.85	5.64	4.34	0.685	0.0728	0.0517	0.4106
Catalase, U/mg protein	1102	680	416	420	74.5	0.0574	**0.0002**	0.0530
Total SOD, U/mg protein	46.52	51.26	52.7	50.6	1.342	0.6325	0.322	0.2216
Mn SOD, U/mg protein	22.84	21.07	24.78	20.83	0.935	0.1451	0.6579	0.5718
Cu–Zn SOD, U/mg protein	23.67	30.19	27.92	29.76	1.088	0.0562	0.3692	0.2731
GSHPX, U/mg protein	13.27	8.05	11.06	20.68	2.491	0.6603	0.3041	0.1487

*TG* triglycerides, *PAB* prooxidative-antioxidative balance, *HKU* Hamidi-Koliakos units, *TBARS* thiobarbituric acid reactive substances, *SOD* superoxide dismutase, *GSHPX* glutathione peroxidase.

Significant values are in bold.

**Fig. 2:**
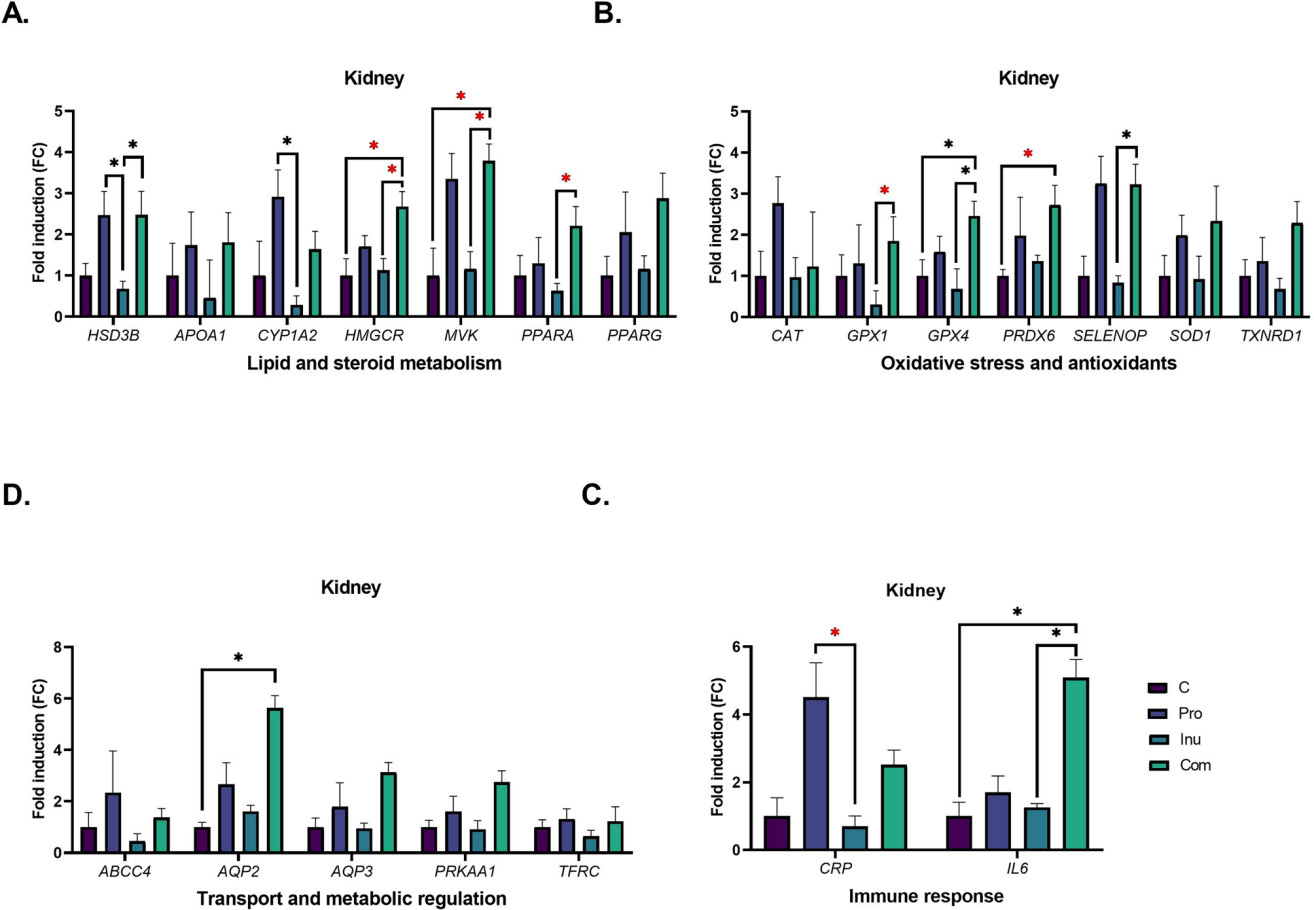
Expression of genes related to energy metabolism, stress response and cellular defence and water homeostasis in the kidneys of growing pigs fed diets supplemented with probiotic (Pro), prebiotic (Inu) or their combination (Com). Data are presented as mean ± SEM. A black asterisk (*) indicates a significant difference between groups (*p* ≤ 0.05). A red asterisk denotes a statistical trend (0.05 < *p* < 0.099).

Supplementation with probiotic did not affect plasma or hepatic and renal lipid profiles. However, animals fed the probiotic-enriched diet showed upregulation of the hepatic *APOA1* gene and a tendency toward higher expression (*p* < 0.1) of peroxisome proliferator-activated receptor alpha (*PPARA*) and ATP binding cassette subfamily C member 4 (*ABCC4*; Fig. 1) compared to the control animals. In addition, probiotic supplementation significantly increased the expression of *HSD3B, BAAT* and *CYP1A2* genes in the kidneys (Fig. 2) compared with pigs fed the prebiotic diet.

Concurrent administration of both probiotic and inulin increased TC and reduced TG levels in the liver (Table 3), accompanied by a trend toward upregulation of *PPARA* in the organ (Fig. 1). Meanwhile, in the kidneys, simultaneous administration of both supplements induced a tendency toward higher expression of the 3-hydroxy-3-methylglutaryl-CoA reductase (*HMGCR*) and mevalonate kinase (*MVK*) genes compared to the control group (Fig. 2). The same trend for renal *HMGCR, MVK* and *PPARA* gene expression was observed in pigs on a diet supplemented with both prebiotic and probiotic when relative to those receiving inulin alone (Fig. 2).

### Mineral status

Inulin supplementation significantly increased plasma sodium and phosphorus concentrations while reducing liver magnesium and copper contents (Table 4). It also upregulated renal aquaporin 2 (*AQP2*) compared to the control group (Fig. 2). On the other hand, dietary probiotic administration elevated renal selenium content (Table 4). A significant interaction between the prebiotic and probiotic was observed for plasma iron and renal phosphorus levels (Table 4). Additionally, probiotic addition to the control diet increased blood iron and renal phosphorus concentrations compared to the unsupplemented diet, whereas supplementation of the inulin diet with probiotic reduced these parameters relative to pigs fed inulin alone.

**Table 4:** Mineral content in the blood plasma, liver, and kidneys of pigs fed the control diet (C) or diets supplemented with inulin (Inu), probiotic (Pro) or their combination (Com).

	Group					*p*-value		
	C	Pro	Inu	Com	SEM	Probiotic	Inulin	Interaction
Blood plasma								
Na, mmol/L	131.52	131.09	133.19	135.62	0.723	0.4714	**0.0313**	0.3034
K, mmol/L	6.27	6.53	6.12	6.16	0.109	0.6749	0.9742	0.5589
Cl, mmol/L	121	124	122	121	2.0	0.8438	0.7985	0.6778
Mg, mmol/L	0.78	0.84	0.82	0.82	0.016	0.4005	0.7283	0.3949
Ca, mmol/L	2.79	2.90	2.94	2.94	0.031	0.3806	0.1113	0.3451
Zn, µmol/L	12.47	14.16	13.97	14.03	0.296	0.1347	0.2378	0.1580
Cu, µmol/L	24.73	24.99	24.58	25.58	0.633	0.6389	0.8685	0.7848
Fe, µmol/L	30.84	39.49	34.49	26.96	1.601	0.8481	0.1363	**0.0094**
Mn, nmol/L	7.04	6.78	6.61	6.52	0.539	0.8740	0.7644	0.9425
Se, nmol/L	159.6	189.0	196.6	199.5	10.3	0.4428	0.2626	0.5296
P, mmol/L	2.94	2.97	3.09	3.24	0.053	0.3955	**0.0478**	0.5675
Liver (mg/kg wet tissue)								
Na	716	736	669	713	12.8	0.2166	0.1779	0.6261
K	2168	2110	2155	2181	15.0	0.6003	0.3463	0.1697
Mg	187	184	178	182	1.4	0.8777	**0.0429**	0.2211
Ca	41.72	42.45	40.37	41.90	0.489	0.2650	0.347	0.6917
Zn	103	106	103	85	5.0	0.4762	0.3095	0.2863
Cu	13.40	10.41	8.53	8.03	0.614	0.0981	**0.0015**	0.2330
Fe	100	96	96	69	8.8	0.3983	0.4016	0.5088
Mn	3.12	2.85	2.85	2.62	0.07	0.0768	0.0716	0.8837
Se	0.085	0.082	0.078	0.082	0.0019	0.9428	0.334	0.4211
P	2075	2237	2014	1997	51.0	0.4869	0.1533	0.3876
Kidney (mg/kg wet tissue)								
Na	1035	988	980	1087	20	0.4478	0.5692	0.0599
K	1864	1980	1883	1894	24.5	0.2196	0.5098	0.304
Mg	180	187	191	185	2.1	0.8568	0.3382	0.1523
Ca	59.26	63.05	68.08	65.00	1.6	0.9129	0.1044	0.2915
Zn	22.89	24.61	26.57	22.96	0.788	0.5541	0.5248	0.1033
Cu	10.05	10.19	10.57	9.02	0.727	0.6539	0.8343	0.5902
Fe	24.78	22.56	21.23	23.03	0.789	0.896	0.3492	0.2247
Mn	1.66	1.40	1.38	1.38	0.047	0.1582	0.0897	0.1564
Se	0.179	0.202	0.196	0.204	0.0037	**0.0348**	0.1821	0.3003
P	1601	1709	1676	1609	19.1	0.5908	0.7368	**0.0274**

Significant values are in bold.

### Redox balance and antioxidant response

Dietary prebiotic supplementation reduced CAT activity in the blood plasma (Table 2), liver and kidneys (Table 3) compared to the control group. Probiotic supplementation also led to a significant decrease in plasma TBARS levels and CAT activity (Table 2) relative to unsupplemented diets. Pigs receiving both inulin and probiotic had a higher expression of glutathione peroxidase 4 (*GPX4)* and a tendency toward increased *PRDX6* expression in the kidneys compared with controls (Fig. 2). In pigs on the combined diet, renal expression of selenoprotein P (*SELENOP*) and *GPX4* was higher than in the inulin-only group. Concurrent supplementation with inulin and probiotic also tended to increase *GPX1* expression in the kidneys compared with inulin alone.

### Inflammatory markers

Feeding inulin-enriched diets increased plasma fibrinogen level (Table 2). In pigs receiving the combined prebiotic and probiotic supplementation, renal expression of the interleukin-6 (*IL-6*) gene was higher than in the control and inulin-only groups (Fig. 2). In animals fed the probiotic diet, tendency toward upregulation of the C-reactive protein (*CRP*) gene was observed compared to pigs on the inulin diet. Detailed delta threshold values for all analysed genes are presented in Supplementary Table 3.

## Discussion

Previous results showed that neither the addition of prebiotic or probiotic, nor their combination, affected body weight gain or feed intake of piglets from weaning to the end of the trial^17^. Currently, it was shown that the experimental treatments also did not exert any negative effects on liver function in the same animals. This is supported by albumin and total protein concentrations, as well as the reduced activity of liver enzymes in blood. Both total protein and albumin concentrations in blood are well-established indicators of liver function and nutritional status^43^. In the current study, inulin supplementation significantly increased total protein in plasma without adversely affecting albumin levels, which is consistent with our previous observations in pigs^14^. Elevated plasma ALP, AST, and ALT activities are common markers of hepatic dysfunction, disease, or cellular damage^43^. Therefore, the reduced activity of AST and ALT observed following probiotic or combined pre- and probiotic supplementation indicates that these supplements are safe and may support liver health. Our observations align with those of Londono-Perez and Parra-Suescun^44^, who reported decreased liver enzyme activity in post-weaning pigs fed a probiotic-supplemented diet. Additionally, a significant effect of interaction between inulin and probiotic on blood urea concentration was observed, suggesting improved protein digestion in animals receiving both supplements. Probiotics and beneficial gut bacteria are known to enhance protein digestion by secreting proteolytic enzymes that complement the host’s endogenous enzymes^45^. Increased protein digestibility may, in turn, lead to higher hepatic urea synthesis^46^.

Prebiotics, probiotics, and the preparation of both substances can also influence host energy metabolism^47,48^. In our experiment, inulin supplementation significantly increased blood plasma TC and LDL-C levels. However, their concentrations remained within the lower reference range for pigs^49^. Effects of prebiotics on cholesterol metabolism in pigs are inconsistent. Several studies reported that inulin and/or inulin-rich plants reduced blood plasma TC^10,50–52^ and LDL-C^50–52^ levels, while others showed no significant changes^53,54^. Wang et al.^55^ demonstrated that inulin supplementation increased plasma LDL-C, insulin-like growth factor-1, and insulin levels, along with improved carcass traits. The latter authors attributed these effects to improved nutrient utilisation in growing pigs, which may also explain the results observed in the present study.

Inulin and its combination with probiotic significantly affected liver cholesterol levels, albeit in opposite directions. Inulin reduced hepatic cholesterol concentration, while the combined treatment increased it. These differences were not associated with alterations in the expression of liver genes involved in the cholesterol synthesis, specifically *HMGCR* and *MVK*. Similar findings were reported in mice, where inulin modified hepatic cholesterol concentration without affecting the expression of cholesterol synthesis genes^56^. Both prebiotic and probiotic supplementation elevated *APOA1* expression in the liver. The product of this gene plays a key role in reverse cholesterol transport to the liver and is essential for HDL particle formation and maturation^57^. Previously, it was demonstrated that dietary inulin and inulin-rich chicory root stimulate hepatic *APOA1* expression and its serum levels, without significant changes in plasma HDL-C concentrations^11,53,54^. Although an increase in liver and plasma ApoA1 levels would typically be expected to raise HDL-C levels, the cholesterol-to-protein ratio in HDL particles is not constant. An increase in the aforementioned ratio is considered favourable and beneficial for health^58^. Therefore, the upregulation of *APOA1* in pigs may reflect alterations in HDL particle composition and an improvement in the HDL-C/ApoA1 balance.

SCFAs produced by gut bacteria play a crucial role in regulating host’s energy metabolism by modulating genes targeted by peroxisome proliferator-activated receptors (PPARs)^59^. Previously, it was demonstrated that pigs on probiotic-supplemented diets had greater acetic acid concentration in the caecum^17^. By stimulating *PPARα* expression, this SCFA induces fatty acid β-oxidation in the liver^60^. In this study, there was a trend toward upregulation of *PPARA* in the liver and kidney of the same pigs. This is consistent with findings showing that *Lactobacillus*-enriched diets alleviate symptoms of metabolic syndrome through PPARα activation, thereby promoting hepatic fatty acid β-oxidation^61^.

Numerous studies have reported that prebiotic-rich plants, their metabolites, but also probiotics can reduce boar taint^62–65^. The suggested mechanisms involve enzymatic degradation of the pheromone androstenone and the metabolite skatole, processes that occur mainly in the liver^66^. However, in the liver of pigs, no significant differences were detected in the expression of the *HSD3B* and *CYP1A2* genes, which are involved in these pathways. In contrast, both genes were upregulated in the kidneys of pigs fed the probiotic-supplemented diet, and *CYP1A2* expression was also higher in pigs receiving combination of both supplement when compared to inulin fed animals. Apart from the liver, *HSD3B* is expressed in the kidneys, which are also involved in steroid degradation^67^. The skatole-degrading enzyme system involving *CYP1A2* is likewise active in the kidneys^68^. In line with the findings of Rasmussen et al.^63^, who observed *HSD3B* upregulation in the liver of pigs fed inulin-rich dried chicory root, we speculate that microbial activity may contribute to the increased renal expression of this gene observed in our study. A similar explanation is plausible for *CYP1A2*, since dietary supplementation with *L. casei* induced *CYP450* gene expression in the tissues of rats^62^.

Prebiotics, probiotics, and their combination affect mineral absorption from the gastrointestinal tract^69,70^. Sodium absorption in the colon depends on Na/H exchangers, which are strictly regulated by SCFAs such as acetate, butyrate, and propionate in a time- and concentration-dependent manner^71^. The elevated Na^+^ levels in blood plasma of pigs fed diets with inulin partially overlap with the expression profile of AQP2 in the kidneys. This AQP is involved in vasopressin-dependent water reabsorption in the renal collecting ducts (CD) of pigs^72^. Our observations are consistent with previous studies showing increased AQP2 expression and its translocation to the apical domain of basal epithelial cells in the CD of animals given diets supplemented with inulin, with inulin and probiotic, and with inulin-rich chicory root^73,74^. Additionally, increased sodium deposition in the liver and kidneys was found in pigs fed diets supplemented with inulin or chicory root^15,16^. Inulin increased phosphorus concentrations in both blood plasma and kidneys. This is likely due to improved digestibility of inorganic phosphorus induced by bacteria with phytase activity^75^. Our earlier experiment also showed increased phosphorus accumulation in the liver and kidneys after inulin intake^15^. Jolliff and Mahan^76^ reported that inulin supplementation reduced urinary phosphorus excretion, suggesting its higher retention in the body. Lepczyński et al.^14^ have proposed that ITFs in the diet stimulate calcium absorption and promote bone anabolism in growing pigs. This aligns with the current findings regarding elevated blood plasma calcium levels and renal calcium accumulation in pigs fed an inulin-supplemented diet. Previous studies have confirmed that beneficial gut bacteria play a crucial role in Se metabolism by improving its bioavailability^77^. Moreover, selected probiotic strains can also convert inorganic selenium to organic forms incorporated into amino acids^78^. These mechanisms contribute to increased selenium absorption from the gastrointestinal tract and its utilisation for selenoprotein synthesis^79^. This may explain greater levels of selenium and expression of genes encoding selenoproteins in the kidney of pigs fed probiotic-supplemented diets.

The increased selenium bioavailability may partly explain the reduced oxidative stress in animals supplemented with probiotic and the combination of probiotic with inulin, as evidenced by lower blood plasma TBARS levels. Selenoproteins, such as thioredoxin reductases, glutathione peroxidases, and Se-binding proteins like SELENOP, are crucial for maintaining redox homeostasis^80^. Additionally, probiotics can influence several oxidative stress-limiting mechanisms, including increased activity of antioxidant enzymes^81^, induction of reactive oxygen species scavengers in host tissues such as the peroxiredoxins systems^82^, and stimulation of antioxidant metabolite synthesis^83^. These mechanisms seemed to be activated in our experiment as well, as shown by significant changes in selenoprotein expression patterns and a trend toward the upregulation of *GPX1* and *PRDX6* genes in the kidneys of animals of the combined group. We also documented a clear effect of prebiotic administration on oxidative stress biomarkers in animals fed an inulin-supplemented diet. In this group, TBARS concentrations in the liver and kidneys were more than twofold lower than in the control one. While the effect of inulin on plasma TBARS showed only a decreasing trend, it was accompanied by a significant reduction in CAT activity in both organs. These observations are consistent with our previous reports, which clearly demonstrated the antioxidant effects of inulin in growing pigs^10,15^. Moreover, it should be noted that dietary prebiotic, probiotic and their simultaneous administration significantly reduced CAT activity in the blood plasma of pigs, indicating lower oxidative stress as a result of these interventions.

Surprisingly, dietary supplementation with the mixture of inulin and probiotics increased the expression of the *IL-6* gene in the kidneys, while inulin alone increased the blood plasma fibrinogen concentration. This contrasts with our recent study^84^, where a 40-day supplementation with 3% native chicory inulin led to a marked decrease in plasma expression of fibrinogen beta chain protein in growing pigs. We may speculate that the mechanisms responsible for phenomenon observed in our study may be related to the microbial-associated molecular patterns (MAMPs) induction by both probiotics and prebiotics and have a physiological nature. It is known that probiotic bacteria i.a. *Lactobacilli* are stimulators of pattern recognizing receptors and/or toll-like receptors^85^, thus its direct supplementation or stimulation of beneficial bacteria growth or metabolic activity by the fermentation of inulin may activate innate immune response mechanisms including IL-6 and other cytokines upregulation and transient stimulation of acute phase proteins synthesis. Moreover, MAMPs bioactivity is highly strain specific, and even subtle genomic or regulatory differences may drive divergent immunomodulatory effects, thus it complicates the measurement of specific outcomes, also a supplementation type or level, microbiota composition and animals’ physiological status may be a factors shaping the specific immune response^86^. Nevertheless, most of the research points out anti-inflammatory properties of probiotic bacteria^87^ and prebiotics^88^. Thus, phenomenon observed in our study may be a promising target for further research especially in animals in early post weaning period where marked changes in intestinal ecology occurs.

## Conclusions

In summary, both supplements, either applied separately or in combination were safe and supported favourable physiological responses in pigs.

Inulin supplementation promoted liver protein synthesis and turnover, as reflected by elevated plasma total protein concentrations. It also significantly affected lipid metabolism by increasing plasma total and LDL cholesterol levels, while reducing hepatic total cholesterol and renal TG concentration. These effects were accompanied by higher hepatic *APOA1* expression, indicating more efficient lipid transport and cholesterol efflux. Inulin also reduced catalase activity in the plasma, liver, and kidneys, which may suggest reduced oxidative stress mediated by inulin-derived microbial metabolites.

Probiotic supplementation improved hepatic enzyme homeostasis, as evidenced by reduced plasma AST and ALT activity. It also attenuated oxidative stress and lipid peroxidation, demonstrated by lower PAB and TBARS levels, and CAT activity in blood plasma. Probiotic intake stimulated the expression of genes involved in lipid metabolism (*APOA1, PPARA, ABCC4*) in the liver and steroid metabolism (*HSD3B, CYP1A2*) in the kidneys, indicating improved metabolic function.

Feeding both supplements simultanously increased hepatic total cholesterol and reduced triglyceride concentrations. These changes coincided with a tendency for higher expression of genes regulating lipid metabolism ( *HMGCR*, *MVK*) in the kidneys and *PPARA* in the liver. Furthermore, the combined supplementation activated renal mechanisms preventing oxidative stress, as measured by the increased expression of the *GPX4* and *PRDX61* genes.

## Supplementary Information

Below is the link to the electronic supplementary material.

Supplementary Material 1

## Data Availability

None of the data have been deposited in an official repository. Data supporting the findings are available from the authors upon request.
