# Supplementary material for: Inulin and multispecies probiotic effects on blood, liver and kidney biochemistry and metabolic and stress-related gene expression in pigs
Source: Sci Rep. 2026 Mar 13;16:13343. doi: 10.1038/s41598-026-43434-7 (PMC13106808; doi:10.1038/s41598-026-43434-7)
Supplement: Supplementary file 1 — Supplementary Material 1 [file 41598_2026_43434_MOESM1_ESM.docx]

|  | Diet | | | |
| --- | --- | --- | --- | --- |
| mg/kg | C | Pro | Inu | Com |
| Mg | 2301,3 | 2308,1 | 2292,5 | 2309,1 |
| Ca | 10768 | 10738 | 10934 | 10899 |
| Na | 2842,7 | 2875,8 | 2926,2 | 2875,6 |
| K | 9107 | 9566 | 9319 | 9606 |
| Zn | 304,58 | 327,70 | 318,71 | 324,51 |
| Cu | 43,133 | 43,137 | 43,551 | 43,165 |
| Fe | 799,9 | 759,1 | 764,2 | 806,7 |
| Mn | 148,90 | 146,88 | 148,48 | 144,09 |
| Cr | 2,400 | 2,049 | 2,274 | 2,087 |
| Pb | 3,569 | 3,333 | 3,479 | 3,633 |
| Cd | 0,037 | 0,031 | 0,033 | 0,036 |
| Se | 15,444 | 15,249 | 15,414 | 15,271 |
| P | 7445 | 7529 | 7580 | 7451 |
| Al | 227,66 | 206,74 | 211,85 | 228,45 |
| Si | 2922,72 | 2922,12 | 2926,19 | 2820,20 |

**Supplementary Table** **1** Macro- and micromineral content in the pig diets: control diet (C), diets supplemented with 0.05 % of probiotics (Pro), 2 % of inulin (Inu) or with 2 % of inulin and 0.05 % of probiotics (Com) (mg/kg)

**Supplementary Table** **2** Reference material - bovine muscle 8414

| Element |  | Concentration of elements in reference material 8414 (mg/kg) | | | | | Fold change observed result/  certified result |
| --- | --- | --- | --- | --- | --- | --- | --- |
|  | Certified  result | | 1st replicate | 2nd replicate | 3rd  replicate | Observed result |  |
| Pb | 0.38 | | 0.37810 | 0.39030 | 0.3855 | 0.38460 | 1.0121 |
| Cd | 0.013 | | 0.01412 | 0.01403 | 0.14001 | 0.01405 | 1.0807 |
| Zn | 142 | | 144.9548 | 143.9961 | 141.5583 | 143.50306 | 1.0105 |
| Cu | 2.84 | | 2.85051 | 2.83415 | 2.86014 | 2.48266 | 1.0029 |
| Mn | 0.37 | | 0.39025 | 0.36531 | 0.38221 | 0.37925 | 1.0250 |
| Cr | 0,071 | | 0.06804 | 0.069954 | 0.06889 | 0.068961 | 0.9712 |
| Fe | 71.2 | | 69.71051 | 70.92395 | 74.00512 | 71.54652 | 1.0048 |
| Al | 1.7 | | 1.66991 | 1.61889 | 1.68229 | 1.65703 | 0.9747 |
| Ca | 145 | | 146.9942 | 149.07511 | 145.92551 | 147.33160 | 1.0160 |
| Se | 0.076 | | 0.07271 | 0.069991 | 0.072991 | 0.0718973 | 0.9460 |
| K | 15170 | | 15381.0 | 15221.50 | 15255.43 | 15318.215 | 1.0097 |
| Na | 2100 | | 2055.150 | 2115.93 | 2075.95 | 2082.3433 | 0.9915 |
| Mg | 960 | | 971.440 | 963.451 | 954.89 | 963.260333 | 1.0033 |
| P | 8360 | | 8299.05 | 8315.991 | 8285.51 | 8300.1836 | 0.9928 |

**Supplementary Table 3.** Effect of pig dietary supplementation on gene expression (delta threshold cycle, ∆Ct) in liver and kidney tissue.

|  | Group | | | |  |  |  |  |
| --- | --- | --- | --- | --- | --- | --- | --- | --- |
|  | C | Pro | Inu | Syn |  | SEM |  | *p*-value |
| ***Liver*** |  |  |  |  |  |  |  |  |
| Lipid and steroid metabolism | | | | | | | | |
| *HSD3B* | 8.57 | 6.76 | 7.77 | 7.62 |  | 0.327 |  | 0.3402 |
| *APOA1* | 7.42^a^ | 4.01^b^ | 4.52^b^ | 5.15 |  | 0.449 |  | **0.0431** |
| *BAAT* | 11.68 | 8.69 | 10.30 | 10.13 |  | 0.557 |  | 0.1371 |
| *CYP1A2* | 8.18 | 5.13 | 6.57 | 6.93 |  | 0.561 |  | 0.5479 |
| *HMGCR* | 5.32 | 3.11 | 4.10 | 3.92 |  | 0.406 |  | 0.4515 |
| *MVK* | 7.90 | 6.24 | 6.54 | 7.22 |  | 0.459 |  | 0.3030 |
| *PPARA* | 4.02^a^ | 1.59^b^ | 2.92 | 2.13^b^ |  | 0.919 |  | **0.0788** |
| *PPARG* | 10.66 | 8.40 | 9.42 | 10.11 |  | 0.323 |  | 0.1206 |
| Oxidative stress and antioxidants | | | | | | | | |
| *CAT* | 2.48 | -0.47 | 1.19 | 1.47 |  | 0.458 |  | 0.2115 |
| *GPX1* | 9.50 | 7.95 | 8.48 | 8.73 |  | 0.319 |  | 0.4320 |
| *GPX4* | 6.29 | 4.81 | 5.60 | 5.74 |  | 0.279 |  | 0.4515 |
| *PRDX6* | 8.12 | 6.64 | 7.62 | 8.09 |  | 0.286 |  | 0.2075 |
| *SELENOP* | 2.41 | 0.52 | 1.08 | 1.28 |  | 0.373 |  | 0.4515 |
| *SOD1* | 6.36 | 5.10 | 6.39 | 5.58 |  | 0.285 |  | 0.4023 |
| *TXNRD1* | 7.35 | 4.49 | 5.70 | 6.14 |  | 0.424 |  | 0.1108 |
| Transport and metabolic regulation | | | | | | | | |
| *ABCC4* | 7.58^a^ | 5.68^b^ | 8.03 | 8.48 |  | 0.386 |  | **0.0695** |
| *PRKAA1* | 4.80 | 3.44 | 4.41 | 4.15 |  | 0.292 |  | 0.5208 |
| *TFRC* | 4.34 | 2.32 | 3.32 | 3.53 |  | 0.329 |  | 0.3002 |
| Immune response | | | | | | | | |
| *CRP* | 7.04 | 3.96 | 5.46 | 5.64 |  | 0.490 |  | 0.2464 |
| *IL6* | 7.63 | 6.78 | 7.43 | 7.49 |  | 0.201 |  | 0.5208 |
| ***Kidney*** |  |  |  |  |  |  |  |  |
| Lipid and steroid metabolism | | | | | | | | |
| *HSD3B* | 12.98 | 11.67^ac^ | 13.52^b^ | 11.67^ac^ |  | 0.269 |  | **0.0154** |
| *APOA1* | 12.61 | 11.81 | 13.72 | 11.76 |  | 0.404 |  | 0.3795 |
| *CYP1A2* | 15.06 | 13.51^a^ | 16.84^b^ | 14.34 |  | 0.379 |  | **0.0212** |
| *HMGCR* | 10.91^a^ | 10.14 | 10.74^a^ | 9.49^b^ |  | 0.195 |  | **0.0683** |
| *MVK* | 15.44^a^ | 13.70 | 15.23^a^ | 13.52^b^ |  | 0.311 |  | **0.0575** |
| *PPARA* | 8.21 | 7.83 | 8.88 | 7.07 |  | 0.256 |  | **0.0916** |
| *PPARG* | 12.57 | 11.54 | 12.36^a^ | 11.05^b^ |  | 0.391 |  | 0.3694 |
| Oxidative stress and antioxidants | | | | | | | | |
| *CAT* | 8.43 | 6.95 | 8.46 | 8.13 |  | 0.395 |  | 0.4476 |
| *GPX1* | 14.69 | 14.29 | 16.37^a^ | 13.79^b^ |  | 0.368 |  | **0.0694** |
| *GPX4* | 10.81^a^ | 10.15 | 11.38^a^ | 9.51^b^ |  | 0.247 |  | **0.0488** |
| *PRDX6* | 12.83^a^ | 11.84 | 12.39 | 11.39^b^ |  | 0.269 |  | **0.0963** |
| *SELENOP* | 6.87 | 5.17 | 7.14^a^ | 5.18^b^ |  | 0.299 |  | **0.0123** |
| *SOD1* | 10.21 | 9.22 | 10.33 | 8.99 |  | 0.305 |  | 0.3480 |
| *TXNRD1* | 11.76 | 11.32 | 12.32 | 10.57 |  | 0.248 |  | 0.1236 |
| Transport and metabolic regulation | | | | | | | | |
| *ABCC4* | 9.61 | 8.38 | 10.74 | 9.14 |  | 0.440 |  | 0.1581 |
| *AQP2* | 11.02^a^ | 9.61 | 10.34 | 8.53^b^ |  | 0.307 |  | **0.0340** |
| *AQP3* | 13.54 | 12.70 | 13.61 | 11.90 |  | 0.284 |  | 0.1989 |
| *PRKAA1* | 9.16 | 8.47 | 9.28 | 7.69 |  | 0.237 |  | 0.1212 |
| *TFRC* | 11.20 | 10.80 | 11.82 | 10.91 |  | 0.195 |  | 0.2488 |
| Immune response | | | | | | | | |
| *CRP* | 15.89 | 13.72^a^ | 16.40^b^ | 14.55 |  | 0.381 |  | **0.0591** |
| *IL6* | 13.63^a^ | 12.86 | 13.29^a^ | 11.28^b^ |  | 0.259 |  | **0.0311** |

^a, b, c^ - Superscripts indicate differences between groups in which the effect of a factor in the Kruskal-Wallis test was at the level of 0.05<*p*<0.099. Intergroup comparisons were performed using Dunn's test.
